# Unraveling epigenetic drivers of immune evasion in gliomas: mechanisms and therapeutic implications

**DOI:** 10.3389/fimmu.2025.1633338

**Published:** 2025-08-25

**Authors:** Dan Wu, Dongen Ju, Yujia Zhao, Wenna Liu, Qingqing Liu, Ying Liang

**Affiliations:** ^1^ Precision Pharmacy and Drug Development Center, Department of Pharmacy, Tangdu Hospital, Fourth Military Medical University, Xi’an, Shaanxi, China; ^2^ Department of Urology, Xijing Hospital, Fourth Military Medical University, Xi’an, Shaanxi, China; ^3^ Department of Oncology, Tangdu Hospital, Fourth Military Medical University, Xi’an, Shaanxi, China

**Keywords:** gliomas, epigenetic regulations, immune evasion, non-coding RNA, CAR-T cell therapy

## Abstract

Gliomas are the most common primary malignant tumors of the central nervous system (CNS), and despite progress in molecular diagnostics and targeted therapies, their prognosis remains poor. In recent years, immunotherapy has emerged as a promising treatment modality in cancer therapy. However, the inevitable immune evasion by tumor cells is a key barrier affecting therapeutic efficacy. Epigenetic regulation, such as DNA methylation, histone modification, and non-coding RNA expression, plays a crucial role in the occurrence, development, and immune evasion of gliomas. These modifications can dynamically regulate gene expression, leading to the silencing of tumor-associated antigens, dysregulation of pro-inflammatory cytokines, and dynamic modulation of immune checkpoints (such as PD-L1). This review systematically elucidates the key mechanisms by which epigenetic regulation promotes immune evasion in gliomas and details three interconnected mechanisms: 1) epigenetic silencing of tumor-associated antigens and antigen-presenting machinery; 2) dysregulation of pro-inflammatory cytokine secretion; and 3) dynamic modulation of PD-L1 expression through chromatin remodeling. We emphasize the potential of combining epigenetic therapies with immunotherapies to enhance anti-tumor immune responses and overcome treatment resistance in gliomas. Future research should focus on developing biomarker-driven epigenetic immunotherapies and exploring the complex interplay between epigenetic modifications, glioma cells, and the tumor immune microenvironment to improve patient outcomes.

## Graphical Abstract

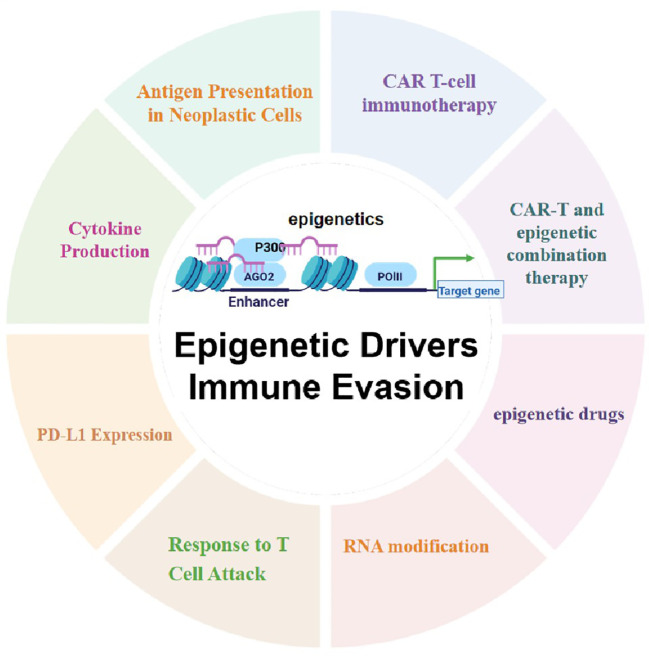

## 1 Introduction

Gliomas represent the preeminent primary tumors of the central nervous system, arising from diverse glial cell lineages such as astrocytes, oligodendrocytes, and ependymal cells, these tumors exhibit a high degree of heterogeneity. According to the molecular complexity and the diversity of pathological morphology, the World Health Organization (WHO) categorizes gliomas into four malignancy grades in ascending order, with Grade IV representing the most aggressive form (1). In the adult population, diffuse infiltrating gliomas are mainly classified into three categories: astrocytoma, oligodendroglioma, and glioblastoma. Among them, astrocytoma patients with IDH mutations exhibit substantially improved survival outcomes compared to those with IDH wild-type glioblastoma. Additionally, oligodendroglioma cases with concurrent IDH mutation and 1p/19q codeletion demonstrate the most promising prognostic profile (2, 3). In the pediatric population, pilocytic astrocytoma is relatively common. This tumor is often accompanied by BRAF gene variation and exhibits a relatively well-circumscribed growth pattern, with an overall good prognosis. On the other hand, diffuse midline glioma with H3K27 variation has the highest degree of malignancy and is a key factor leading to death due to glioma in children (4, 5).

Despite significant advances in understanding glioma pathogenesis, molecular diagnosis, and targeted therapy via omics approaches (e.g., gene sequencing, single-cell and spatial transcriptomics) (6–9), prognosis remains poor. Thus, elucidating pathogenic mechanisms and identifying novel therapies are critical to improving patient outcomes and quality of life. Immunotherapy, including immune checkpoint inhibition and CAR-T therapy, has advanced rapidly in oncology, offering therapeutic promise. However, gliomas exhibit strong immunosuppressive properties: glioma cells upregulate immunosuppressive factors (e.g., PD-L1, IDO) to impair antigen presentation, while glioma-associated macrophages and regulatory T cells weaken immunity via inhibitory cytokine secretion and cytotoxic T lymphocyte depletion, respectively (10). This immunosuppressive microenvironment impairs immunotherapy efficacy. For instance, phase III trials showed that the checkpoint inhibitor nivolumab failed to improve survival in newly diagnosed glioblastoma (with standard treatment) or recurrent cases (vs. bevacizumab) (11). Recently, Claudia Galassi et al. inspired by the “three Es” model (Elimination, Equilibrium, Escape) (12), Galassi et al. proposed a “three Cs” framework (camouflage, coercion, cytoprotection) to explain cancer immune evasion (13): evading immune recognition, interfering with effector cell activation, and resisting cytotoxicity. These mechanisms underpin glioma cells’ ability to escape immune attack, critically contributing to immunotherapy failure. Thus, uncovering novel regulators of glioma immune escape is vital for advancing clinical immunotherapy.

Epigenetic modifications, dynamic chemical alterations of chromosomal nucleic acids/proteins, orchestrate gene expression across splicing, transcription, and chromatin architecture without changing DNA sequence. Regulated by DNA methylation, histone modification, chromatin remodeling, and non-coding RNAs (ncRNAs), they play pivotal roles in tumorigenesis and progression (14). Recent studies show histone methylation/acetylation modulates immune checkpoints (e.g., PD-1/PD-L1), affecting immune recognition and elimination of tumor cells (15, 16). Additionally, ncRNAs regulate immune cell activation via immune checkpoints, epithelial-mesenchymal transition (EMT), and the tumor immune microenvironment (TIME), thereby influencing immunotherapy efficacy (17). These findings highlight the critical role of epigenetic mechanisms in immune cell differentiation and function, dictating context-specific gene expression profiles within the tumor immune microenvironment (TIME) that influence immunotherapy outcomes. Given that both tumor neoantigens and autoantigens are immunogenic, epigenetic regulation controls neoantigen generation and reactivates genes restricted to immune-privileged stages (18, 19). Thus, elucidating epigenetic mechanisms underlying tumor immune escape and developing combinatorial strategies with immunosuppressants and epigenetic drugs hold promise for overcoming immune escape in gliomas.

This review systematically outlines key epigenetic mechanisms underlying glioma immune evasion, including silencing of tumor-associated antigens and antigen-presenting machinery, dysregulated pro-inflammatory cytokine secretion, and dynamic PD-L1 modulation via chromatin remodeling. We also evaluate preclinical/clinical strategies targeting such epigenetic-driven immune evasion, offering a framework for developing biomarker-guided epigenetic immunotherapies to overcome glioma treatment resistance.

## 2 Epigenetics in glioma development and progression

### 2.1 Epigenetic regulation in glioma subtype classification

**Figure 1:**
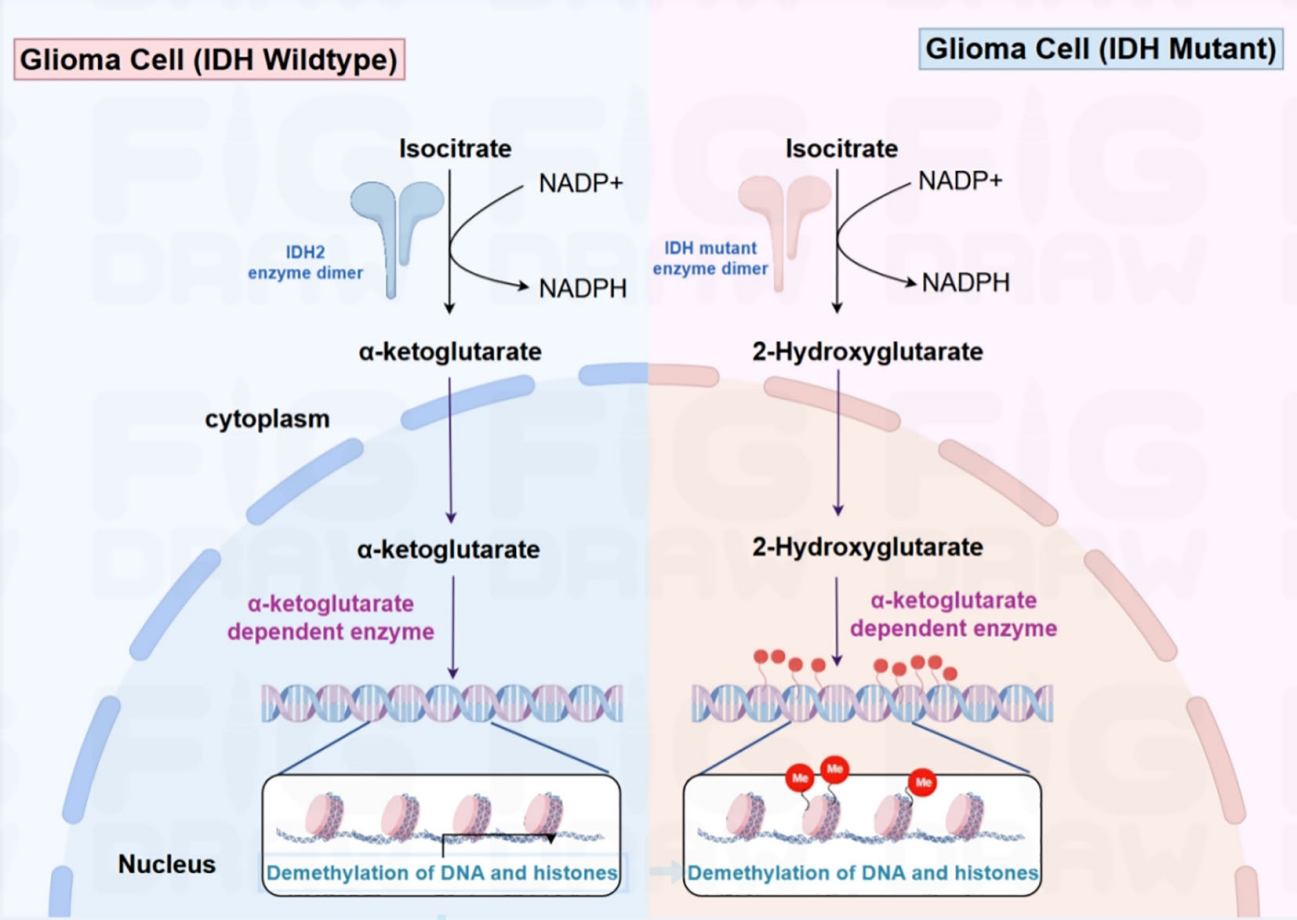
In IDH wild-type glioma cells (left), isocitrate is converted into α-ketoglutarate and NADPH using NADP^+^ under the action of the IDH2 enzyme dimer. α-ketoglutarate then enters the nucleus and participates in α- ketoglutarate-dependent enzymatic reactions, promoting DNA and histone demethylation. In IDH mutant glioma cells (right), the mutated IDH enzyme catalyzes the conversion of isocitrate to 2-hydroxyglutarate (2-HG), leading to intracellular accumulation of this metabolite. By inhibiting the function of α-ketoglutarate-dependent enzymes, 2-HG obstructs DNA and histone demethylation processes, thereby impacting gene expression modulation.

Epigenetic alterations are crucial in determining the classification and driving the progression of gliomas. The genetic alteration status of isocitrate dehydrogenase (IDH) is a critical biomarker that distinguishes different glioma subtypes. As a crucial metabolic enzyme, IDH is mainly involved in the cellular energy metabolism process, especially the tricarboxylic acid cycle (TCA cycle). It plays an important role in maintaining normal physiological functions. In glioma cells, IDH mutation is a common molecular alteration, particularly the IDH1-R132H mutation. This mutation leads to changes in enzyme activity, converting α-ketoglutarate (α-KG) into (R)-2-hydroxyglutarate [(R)-2HG]. This metabolite can interfere with normal cellular metabolism and epigenetic regulation, promoting the occurrence and development of tumors (20) (**Figure 1**).

Interestingly, IDH-mutant gliomas display a CpG island methylator phenotype (G-CIMP), which is characterized by hypermethylation in CpG-rich promoter regions. This epigenetic alteration is associated with distinct clinical outcomes, with the “high” G-CIMP subgroup generally having a better prognosis (21). The dynamic interplay between IDH mutations and epigenetic modifications is further evidenced by the role of G-CIMP in driving the differentiation and aggressiveness of glioma subtypes. For instance, oligodendrogliomas harboring IDH mutation alongside 1p/19q codeletion show a more promising prognosis, whereas glioblastomas with wild-type IDH are associated with a poorer outcome. Compared with untreated patients, patients who experience relapse and exhibit cases with the absence of O(6)-methylguanine-DNA methyltransferase (MGMT) promoter methylation often exhibit a greater load of genetic mutations, indicating that this methylation phenotype may lead to the chemoresistance of tumors (22, 23). These observations underscore the clinical relevance of epigenetic profiling in glioma subtype classification and prognostication.

Even though extensive genomic hypermethylation has been demonstrated to impede the process of cellular differentiation, the role of specific methylation events in tumor progression has been emphasized. This phenomenon involves the transcriptional silencing of tumor suppressor genes (including BRCA1/BRCA2, TP53BP1, SMAD4) and the abnormal activation of oncogenes (such as PDGFRA, PIK3CA, and BRAF), thereby facilitating tumor initiation and progression. These genes are collectively referred to as “methylation-dependent oncogenic drivers” (24). IDH-mutated low-grade gliomas (LGGs) represent a subgroup of brain tumors exhibiting unique molecular features, typically characterized by slow growth and relatively favorable prognosis. However, they carry the potential risk of malignant progression. The progression from low-grade to high-grade malignancy is propelled by the accretion of genetic mutations, epigenetic modifications, and shifts in the tumor microenvironment (TME). Therefore, early identification of the IDH molecular subtypes in glioma patients and targeted treatment have significant clinical implications for patient outcomes. In a recent investigation, Wu and his team constructed a dual-phase framework by probing single-cell RNA sequencing and chromatin accessibility profiles across various glioma grades. During the initial phase, gliomagenesis is driven by oligodendrocyte precursor-like cells in conjunction with epigenetic reprogramming, which deactivate tumor suppressor genes such as CDKN2A and trigger oncogenes like PDGFRA (25). As the disease progresses, tumor expansion is fueled by an increased population of proliferative neural precursor-like cells. Genetic aberrations, including PDGFRA, MYCN, and CDK4 amplifications alongside CDKN2A/B deletions, facilitate tumor advancement. This research underscores the fluctuating impact of IFN signaling during disease progression. In IDH-mutated low-grade gliomas, IFN signaling undergoes epigenetic hypermethylation-mediated suppression, a process reversible by MGMT inhibitors or IDH antagonists, reactivating the IFN pathway. In contrast, high-grade gliomas evade IFN signaling through genetic deletions of IFN genes. These findings indicate a shift from epigenetic to genetic control in glioma progression. Lately, the US Food and Drug Administration (FDA) greenlit vorasidenib, a brain-permeable small molecule targeting mutant IDH1/2 proteins, marking a pivotal change in the therapeutic approach for IDH-mutant gliomas (26).

### 2.2 Epigenetic alterations shaping the transcriptional landscape of glioma

Epigenetic alterations significantly impact the transcriptional landscape of glioma cells, contributing to their malignant behavior. Research has shown that mIDH1 expression induces transcriptional silencing of the PD-L1-encoding gene CD274 via DNA methylation mechanisms (27, 28). Upon treatment with mIDH1 inhibitors, mIDH1 glioma cells exhibit reduced DNA methylation in the CD274 regulatory region. Concurrently, tumor cell-mediated high PD-L1 expression contributes to T cell exhaustion. Therefore, in gliomas with mIDH1 mutations, the concentration of PD-L1, which restricts T-cell functionality, is notably diminished. A separate study revealed that the absence of ATRX-a key epigenetic regulator linked to adult and pediatric gliomagenesis-epigenetically drives the production of PD-L1 alongside multiple immunosuppressive cytokines. This cascade of events ultimately initiates an immune-tolerance-promoting process in ATRX-mutated gliomas (29–31).

Explorations into the influence of histone modifications on TME modulation are insufficiently documented. Emerging findings highlight that RACK7 (encoded by *ZMYND8*) displays intensified interaction with the H3.3-G34R histone variant, a mutation linked to pHGG pathogenesis. This augmented binding event results in silencing of specific genomic loci, including CIITA, which plays a pivotal role in governing MHC class II molecule expression (32). The PRC2 complex, a group of histone methyltransferases, plays an essential part in regulating epigenetic silencing, with its activity being modifiable in gliomas. The expression levels of PRC2 proteins are associated with a poor prognosis (33). Recent investigations demonstrate that PRC2-dependent chromatin silencing contributes to immunosuppressive TMEs by inhibiting the transcription of immunostimulatory cytokines in neoplastic cells. Therefore, tumors with PRC2 mutations respond better to ICIs, providing the possibility of enhancing immunotherapy response by epigenetically and pharmacologically restoring PRC2 methyltransferase activity (34). Besides, in mIDH1-mutant gliomas, epigenetic modulation of the Notch signaling cascade is achieved through DNA methylation at CpG dinucleotides within the delta-like ligand 3 (DLL3) genomic locus. As an inhibitory Notch ligand, DLL3 expression exhibits a positive correlation with survival outcomes in mIDH1 gliomas (35), Glioma cases with elevated DLL3 levels demonstrate enhanced immune cell infiltration, suggesting a functional link between Notch pathway activation and immune responsiveness in these neoplasms.

### 2.3 Non-coding RNA in glioma: epigenetic modulators and beyond

Over the past few decades, it has been revealed that noncoding RNAs, such as microRNAs(miRNAs) and long noncoding RNAs, are crucial in gliomas, where they function as epigenetic regulators among other roles (36), Genomic profiling reveals 27 long non-coding RNAs (lncRNAs) are upregulated and 198 are downregulated in glioblastoma (GBM), underscoring their critical involvement in tumor pathogenesis. Recent findings highlight the role of lncRNAs in modulating GBM cell metabolism. For example, the lncRNA TP53TG1 promotes cell migration and proliferation under glucose-deprived conditions by regulating gene expression of PKM2 and IDH1 in glioma cell cultures (37). Additional research demonstrates that lncRNAs influence glioma biology, progression, and therapeutic responsiveness. For example, HOTAIRM1, an epigenetic modulator, associates with transcriptional start sites to regulate gene expression. LncSNHG6 and lncRNA ZFAT-AS1 facilitate Histone H3 Lysine 27 trimethylation (H3K27me3) deposition, leading to transcriptional silencing. MiRNAs as endogenous regulatory RNAs, function by inhibiting mRNA translation. Depletion of miRNAs that govern key mRNAs- including miR-31 (which targets CDKN2A/B) and miR-34a (involved in EGFR level regulation) (38). T-can drive oncogenic pathways. Variations in miRNA expression across molecular subtypes suggest their role in shaping glioma heterogeneity through transcriptional subtype transitions. MiRNA dysregulates major pathways (TGF-β, PI3K/AKT, EGFR, Notch) in glioma progression likewise. Exosomal miR-148a is related to down-regulated CADM1 expression in GBM samples (39). The antagonistic effect of miR-148a reduces the level of p-STAT3 and subsequently up-regulates the level of the tumor suppressor gene CADM1. In addition, NcRNAs act as intercellular signals; glioma cell-secreted miRNA and lncRNA affect TME behavior. For instance, glioma cell-secreted lncRNA-ATB suppresses miR-204-3p in astrocytes, promoting cell migration. Exosome-secreted lncRNAs have a paracrine effect aiding adaptation and resistance. The ncRNA-mediated communication between glioma cells and TME has therapeutic potential, but its role in TME epigenetic changes, especially in immune cells, requires further study. Furthermore, findings from current research indicate that dysregulated expression of circular RNAs (circRNAs) correlates with glioma initiation and progression (40). Transcriptomic profiling via microarray identified 91 circRNAs with altered expression patterns in glioma tissues compared to matched non-tumor adjacent samples. Notably, elevated expression of circ_0037655 has been shown to promote tumor cell viability and invasive capacity (41, 42). The involvement of noncoding RNAs-including lncRNAs, miRNAs, and circRNAs—in shaping the TME and modulating immune cell functionality broadens therapeutic possibilities. Therapeutic approaches targeting these ncRNAs or their associated regulatory pathways may offer innovative strategies to enhance immunotherapeutic efficacy or circumvent treatment resistance. Nevertheless, the intricate interplay among ncRNAs, TME components, and immune cells requires additional investigation to fully harness their potential as therapeutic targets.

**Figure 2:**
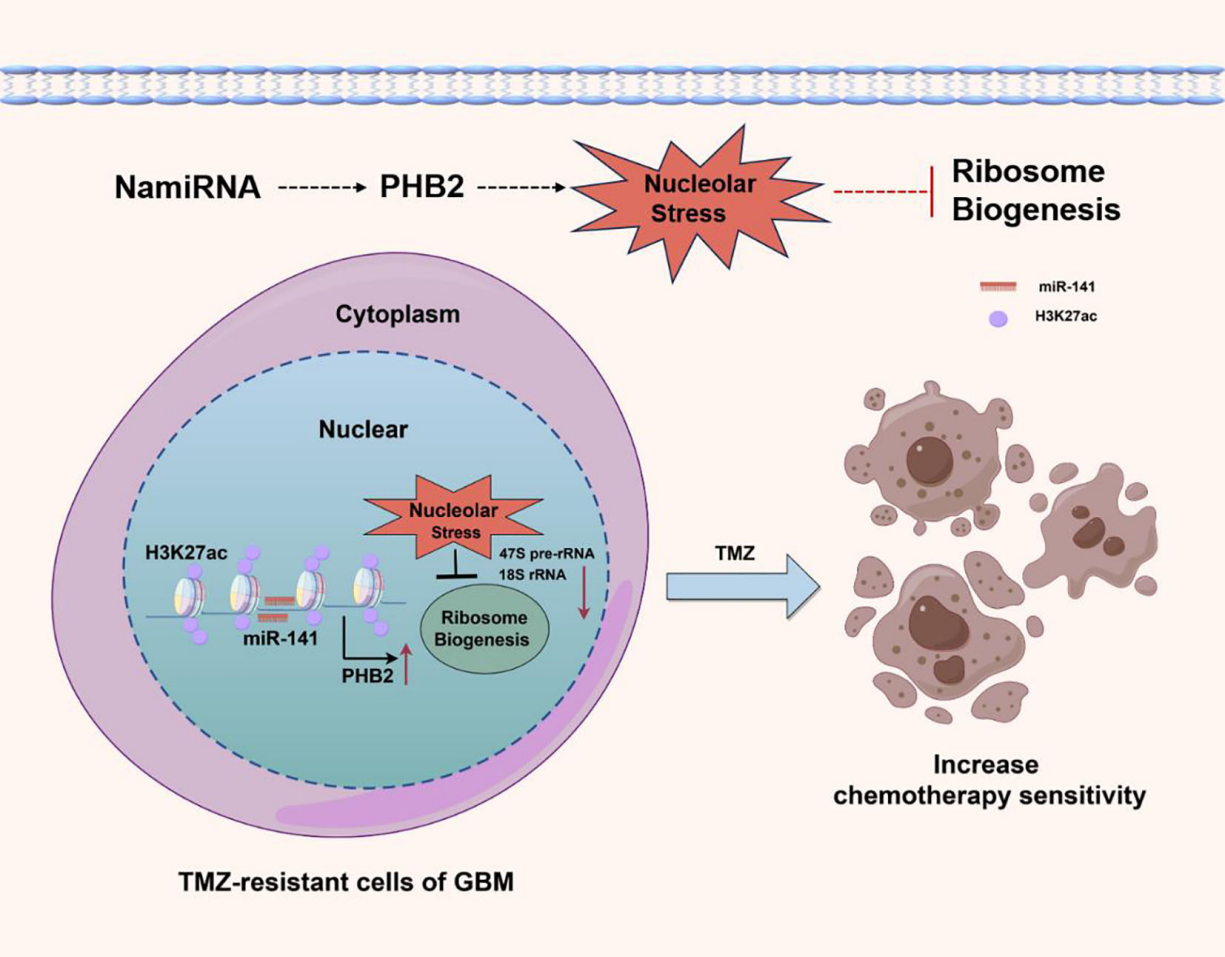
In GBM cells, overexpression of NamiRNA-141 activates enhancer activity, positively regulates PHB2 to induce nucleolar stress, inhibit ribosome biogenesis, and enhance the chemosensitivity of GBM to temozolomide. H3K27ac is an epigenetic marker of active enhancers.

Interestingly, our recent work has revealed a class of miRNAs localized in the nucleus and whose genomic locations highly overlap with enhancers (named NamiRNA, Nuclear Activating miRNA) play a role in enhancing the sensitivity of GBM to temozolomide by activating enhancer activity to positively regulate target genes and induce nucleolar stress (**Figure 2**). This further suggests the clinical application potential of noncoding RNAs in the treatment of gliomas.

### 2.4 Epigenetic regulation and glioma stem cells

**Figure 3:**
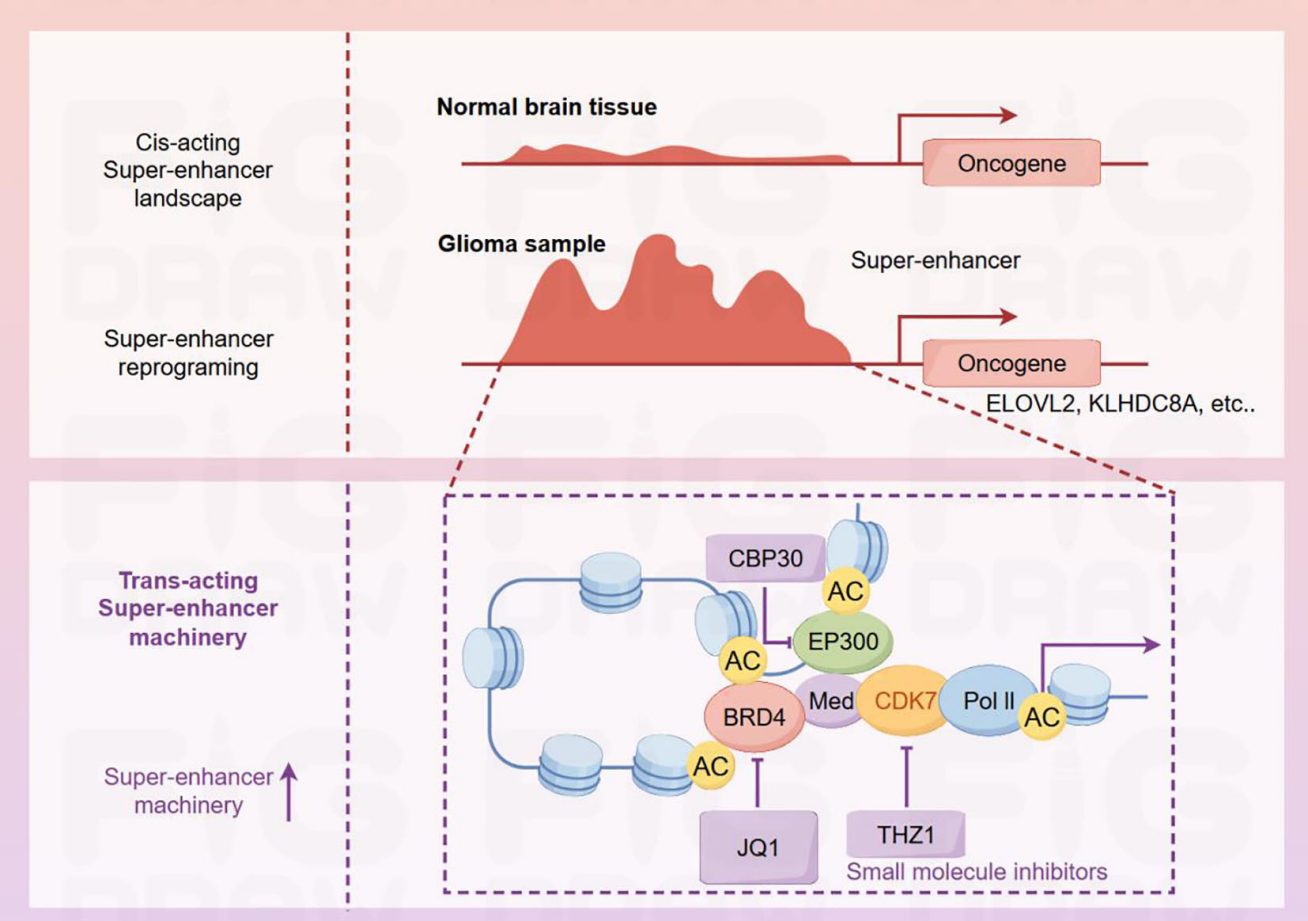
Differences in super-enhancers and related mechanisms of action between normal brain tissues and glioma samples. In normal brain tissues, the cis-acting super-enhancer landscape is relatively stable. In contrast, super-enhancer reprogramming occurs in glioma samples, thereby activating oncogenes such as ELOVL2 and KLHDC8A. The super-enhancer region involves interactions among various proteins, including CBP30, EP300, BRD4, Med, CDK7, and Pol II. The acetylation sites (AC) on these proteins are involved in the regulatory process. The small-molecule inhibitors JQ1 and THZ1 act on BRD4 and CDK7 respectively. By interfering with the super-enhancer machinery, they intervene in the expression of glioma-related genes.

Glioma stem cells (GSCs) are pivotal in driving glioma initiation, progression, and relapse. Their intrinsic resistance to standard therapies and capacity for tumor re-formation pose substantial challenges in glioma management. Emerging research has underscored the complex interplay between epigenetic mechanisms and the preservation of GSC characteristics (43, 44). Epigenetic alterations, such as DNA methylation, histone alterations, and ncRNA-dependent regulation, are central to sustaining the stem-like properties of GSCs (45). For instance, DNA hypermethylation at specific loci can silence tumor suppressor genes, allowing GSCs to evade cellular senescence and apoptosis. Conversely, hypomethylation can activate oncogenes, promoting proliferation and self-renewal (46). Modifications to histones, with a focus on acetylation and methylation, are vital for the upkeep of GSCs. Histone-modifying enzymes, including histone acetyltransferases (HATs) and histone deacetylases (HDACs) manage how accessible chromatin is to transcription factors, which in turn shapes gene expression. High levels of HDAC activity have been observed in GSCs, promoting a repressive chromatin state that favors the expression of stemness-related genes. Inhibitors of HDACs have shown promise in inducing differentiation and reducing the stemness of GSCs, making them more susceptible to conventional therapies. Besides, enhancers are capable of recruiting a large number of transcription factors and coactivators, Histone H3 lysine 27 acetylation (H3K27ac) functions as an indicator of active enhancers and significantly impacts the regulation of genes associated with cell identity and disease. Gimple etc. analyzed 10 glioblastoma specimens obtained via surgical resection and 15 non-neoplastic brain tissue samples through chromatin immunoprecipitation sequencing (ChIP-seq), and screened out the genes WSCD1, ELOVL2 and KLHDC8A that are regulated by glioma stem cell (GSC)-related enhancers, providing new targets for the treatment of gliomas (47) (**Figure 3**). In addition, non-coding RNAs, such as miRNAs and lncRNAs, are increasingly recognized as pivotal modulators of GSC phenotypes. MiRNAs may act as oncogenic or tumor suppressive regulators by targeting genetic loci associated with stem cell maintenance, differentiation, and self-renewal pathways. Analogously, lncRNAs have been demonstrated to modulate gene expression through epigenetic mechanisms, influencing chromatin structure and transcription factor activity. A recent study has indicated that LncRNA INHEG regulates the 2’-O-methylation of rRNA and promotes the translation of mRNA to facilitate the renewal and growth of GSCs (48).

The interplay between epigenetic modifications and GSCs underscores the importance of developing targeted therapies that can disrupt these regulatory networks. By targeting epigenetic modifiers, it may be possible to induce differentiation of GSCs, reducing their tumor-initiating potential and enhancing effectiveness of traditional treatments. Additional investigations into the epigenetic control of GSCs may facilitate the creation of innovative therapeutic approaches to enhance glioma patient outcomes.

## 3 Epigenetic regulation mediated glioma’s immune evasion

Contrasted with other types of cancers, gliomas possess distinct immune-related traits. The anatomical position of gliomas in the brain results in unique attributes that modify the immunological response against neoplastic cells. Gliomas’ unique immunomodulatory characteristics encompass the neuronal and glial cell populations coexist with the BBB. The maintains CNS stability by controlling the passage of substances in and out (49). Although the BBB usually protects the brain from blood toxins while allowing necessary nutrients through, This semi-permeable barrier is also capable of limiting the entry of peripheral leukocytes and therapeutic agents. Brain inflammation impairs BBB integrity, increasing immune cell infiltration, but gliomas may have already escaped immune surveillance by then (50). Epigenetic mechanisms are central to immune evasion in cancer. Within neoplastic cells, histone and DNA modifications can modulate tumor antigen presentation, suppress anti-tumor cytokine expression, and drive PD-L1 checkpoint induction (46).

### 3.1 Antigen presentation in neoplastic cells

Epigenetic control influences the dysfunction of antigen-presenting mechanisms in neoplastic cells, allowing tumors to avoid T-cell recognition and fostering immune evasion (51). For the presentation of self- and tumor-specific peptides to CD8 T cells, tumor cell proteins must undergo proteasomal digestion to produce short oligopeptides. The antigen processing-associated transporters 1 and 2 (TAP1 and TAP2) assemble into a heterodimer, facilitating the translocation of these peptides from the cytosol to the endoplasmic reticulum (ER) (52, 53). Within the ER, antigen peptides are incorporated into nascent major histocompatibility complex class I (MHC-I) molecules with the aid of chaperone proteins (54, 55). The MHC-I complex responsible for tumor antigen presentation consists of two polypeptide subunits-human leukocyte antigens (HLA) and β2-microglobulin (B2M)-and is translocated to the cell surface for antigen presentation. Neoplastic cell MHC-I expression can be suppressed by both DNMT and HDAC enzymes. Administration of DNA methyltransferase inhibitors (DNMTi) or histone deacetylase inhibitors (HDACi) to tumor cells can restore MHC-I surface expression (56). In certain instances, MHC-I deficiency stems from the epigenetic repression of auxiliary genes in the antigen-presentation pathway, including B2M, TAP-1, and TAP-2. Intervention with DNMTi in tumor cell models or clinical settings can upregulate gene expression essential for antigen presentation (57).

Currently, in the context of developing therapies against tumor antigens in gliomas, cancer-specific somatic mutation-derived antigens serve as optimal immunotherapeutic targets from multiple perspectives. Their expression is predominantly restricted to neoplastic cells, thereby significantly mitigating the risk of off-tumor toxicity. For example, in children with diffuse midline glioma (DMG), the H3F3A locus, which encodes histone H3.3, harbors a frequently recurring point mutation (58). The K27M substitution at amino acid position 27 (lysine to methionine) reduces H3K27 trimethylation, causing global demethylation and abnormal gene expression by inhibiting PRC2 activity. This mutation is an attractive immunotherapy target. Analogous to the IDH1 R132H alteration in adult gliomas, the H3.3 K27M mutation exhibits uniform distribution across tumor tissues. Recently, Pavlina Chuntova et al. documented that blocking D-2HG results in the increased expression of proinflammatory genes in a new HLA-A2/HLA-DR1 transgenic mouse model expressing IDH1R132H glioma (59). Meanwhile, Gerard L Brien et al. identified the oncogenic activity in human hindbrain neural stem cells, the histone H3.3-K27M modification arises from the simultaneous impairment of both PRC2 activity and enhancer functionality (60).

Furthermore, in addition to affecting transcription, epigenetic disorders exacerbate genetic instability in gliomas by altering chromatin structure: mutations in histones and ATRX lead to genetic instability due to abnormal deposition of histone marks, while mutant IDH epigenetically enhances DNA damage response signaling. This genetic instability is a key driver of neoantigen production-it not only induces cytoplasmic extrachromosomal DNA to activate the cGAS/STING axis, but also directly causes chromosomal aberrations, prompting mutated proteins to express neoantigens; the immune activation or tolerance triggered by neoantigens is determined by the tumor microenvironment (TME). Epigenetic regulation mediated by STING promoter methylation can modulate glioma immune responses, and this process is reversible by MGMT inhibitors. Mutations in the H3-G34 locus in pediatric high-grade gliomas (pHGG) also induce genomic instability, activate the cGAS/STING axis, enhance immune activation, and improve the efficacy of DNA damage-based therapies due to abnormal neoantigen expression (61, 62).

### 3.2 Cytokine production

Cancer cells can emit signaling molecules that are detected by immune cells at a distance, thereby triggering or inhibiting immune reactions. Glioma cells produce various immunomodulatory factors, such as IL-1β, IL-6, TGF-β, and IL-8. In glioma stem/progenitor-like cells, mIDH1 mediates epigenetic upregulation of G-CSF expression, thereby promoting the remodeling of myeloid cells in the mIDH1-associated TME. Elevated levels of G-CSF stimulate the proliferation of neutrophil precursors and neutrophils, concurrently attenuating the immunosuppressive traits of polymorphonuclear myeloid-derived suppressor cells within the mIDH1-driven tumor microenvironment (22). The epigenetic regulation of tumor cell differentiation impacts the TME. Epigenetic alterations in gliomas, such as H3 mutations in pediatric cases and mIDH1 in adult patients, lead to a halt in cellular differentiation, maintaining cells in a stem-like state. Cells resembling cancer stem cells exhibit reduced immunogenicity and evade immune detection through mechanisms like the downregulation of MHC transcription and the induction of quiescence. A recent investigation revealed that GSCs cultivated in immunocompetent hosts adapt epigenetically and secrete immunosuppressive cytokines (43, 63).

**Figure 4:**
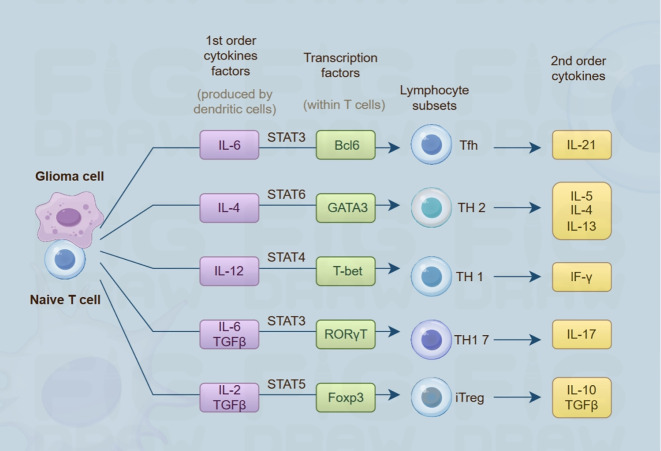
Conceptual illustration of the crosstalk between glioma cell populations and naive T lymphocytes. Glioma-derived cells release primary cytokines (including IL-6, IL-4, IL-12), triggering the activation of cognate transcription factors (such as STAT3, GATA3, T-bet) in naive T cells. These activated transcription factors guide naive T cells to differentiate into different lymphocyte subsets (including Tfh, TH2, TH1, etc.). Subsequently, these subsets produce 2nd-order cytokines (like IL-21, IL-5, IF-γ), which play important roles in the TME and immune regulation.

Furthermore, Glioma cells secrete immunomodulatory cytokines like IL-1β, IL-6, TGF-β, and IL-8 to activate or suppress immune response. Studies show GSCs undergo epigenetic adaptation, secreting immunosuppressive cytokines. Additionally, adapted GSCs upregulate interferon regulatory factor 8 (IRF8) secretion. IRF8, a cytokine usually in myeloid cells, may govern myeloid cell lineages and macrophage polarization (64). This implies that epigenetic mechanisms coordinate the functional repatterning of glioma cell populations, which in turn leads to alterations in immune cells and fosters the development of a TME favorable for tumor growth. Independent research has demonstrated that PRC2 constitutes a complex of histone methyltransferases responsible for epigenetic repression. It is hypothesized that PRC2-dependent chromatin repression facilitates immune evasion by suppressing the production of immunostimulatory cytokines in neoplastic cells (65) (**Figure 4**). Additionally, in gliomas, epigenetic reprogramming of the PI3K/AKT/mTOR signaling axis drives a metabolic shift from oxidative phosphorylation to aerobic glycolysis, and this metabolic reprogramming serves as a core hub for regulating the secretory factor network in the immune microenvironment (66). It exerts its effects through multiple interconnected mechanisms: first, it promotes the massive release of metabolites such as lactate, which not only directly impairs immune cell function and shapes a hypoxia-associated immunosuppressive tumor microenvironment (TME) but also acts as signaling molecules to regulate the expression and secretion of secretory factors; second, it reshapes immune homeostasis via secretory factors through pathways including upregulating TGF-β secretion, inhibiting monocyte differentiation into dendritic cells, and inducing the production of pro-oncogenic cytokines; additionally, metabolic changes in the TME specifically modulate the secretory function of astrocytes, prompting them to release cholesterol to facilitate glioma growth and recruit immunosuppressive macrophages (67). Collectively, these processes reveal that metabolic reprogramming, by systematically regulating the production, release, and function of secretory factors, emerges as a key driver influencing tumor progression and the immune microenvironment.

### 3.3 PD-L1 expression

The interaction between PD-L1 and the immune checkpoint receptor programmed death-1 (PD-1) on T cells conveys inhibitory signals to T cells. This results in the inhibition of T cell proliferation, cytokine generation, and cytotoxic function (68). This phenotype is referred to as “T cell exhaustion”. Consequently, T cells fail to efficiently identify and eliminate tumor cells. This enables tumor cells to dodge the immune system’s monitoring and elimination processes, thereby attaining immune evasion. Epigenetic modulation incontrovertibly assumes a pivotal role in PD-L1 expression. PD-L1 promoter methylation demonstrates an inverse association with PD-L1 transcriptional activity and clinical outcomes in patients (69). Furthermore, HDAC6, which acts as a histone acetylation “eraser,” lysine methyltransferase 2A (KMT2A), a methylation “writer,” and the bromodomain-containing BET family protein BRD4, an acetylation “reader,” each elevate PD-L1 immunoreactivity across melanomas, pancreatic carcinomas, and ovarian neoplasms. As such, targeting these proteins with small-molecule inhibitors can effectively reduce PD-L1 levels and enhance the body’s anti-tumor immune response (70). Additionally, nivolumab is a fully human immunoglobulin G4 monoclonal antibody targeting the programmed death-1 (PD-1) immune checkpoint receptor. Results from a phase III clinical trial (NCT02017717) comparing the efficacy of Nivolumab monotherapy versus bevacizumab in improving survival rates of patients with recurrent glioblastoma showed that the subgroup of glioblastoma patients with methylated MGMT promoters and no baseline corticosteroid dependence may be most likely to benefit from immune checkpoint inhibition (71, 72).

The immune landscape of gliomas is modulated by IDH1/2 genetic alterations, notably, PD-L1 expression is substantially decreased in mutated tumors, providing a mechanistic basis for immune checkpoint inhibitors (ICIs) in IDH1/2 wild-type (wt) patients. Beyond DNA methylation, additional epigenetic regulators may also modulate immune checkpoint (IC) expression in gliomas (73). MiRNAs directly modulate the expression of immune checkpoint regulators, including CTLA-4, PD-1, and PD-L1, via distinct miRNA species. For instance, miR-155 specifically targets CTLA-4, miR-138 fine-tunes both CTLA-4 and PD-1, miR-424 governs PD-L1 and CD80, miR-28 impacts PD-1, while miR-34a, miR-200, miR-513, and miR-138-5p collectively regulate PD-L1 expression (74, 75). Furthermore, overlapping or distinct miRNAs modulate the production of cytokines such as IFN-γ or transcription factors functioning as either activators or repressors of immune checkpoint pathways thereby establishing a redundant and highly intricate regulatory network.

### 3.4 Response to T cell attack

Tumor-infiltrating lymphocytes (TILs) exhibit robust antitumor immune capabilities and represent critical components of immunotherapeutic strategies. In the glioma TME, regulatory T cells (Tregs), CD4^+^ helper T cells (Th cells), and CD8^+^ cytotoxic T cells undergo infiltration. Tregs potently inhibit the functions of antitumor immune cells while elevating the abundance of other immunosuppressive cell types. Helper T cells-particularly Th1 subsets—and CD8^+^ T cells mediate antitumor immunity by respectively enhancing pro-inflammatory responses and executing tumor cell cytotoxicity (76). The efficacy of their antitumor actions is impeded due to limited tumor infiltration and the immunosuppressive milieu of glioma-derived TME. The primary driver of immune suppression is the glioma cells themselves, MDSCs, Tregs, and Tumor-Associated Microglia and Macrophages (TAMMs) through PD-L1 expression and TGF-β/IL-10 secretion. Immune-modulatory molecules trigger T cell functional impairment, encompassing anergy and exhaustion phenotypes. Within exhausted Th and CD8^+^ T cell subsets, proliferative capacity is diminished, and secretion of effector cytokines (e.g., IL-2, TNF-α, IFN-γ) is significantly reduced. Mechanistically, T cell exhaustion is typified by enhanced chromatin openness at immunosuppressive gene loci (PDCD1, CTLA4, LAG3, ENTPD1) and reduced accessibility at cell differentiation gene loci (IL7R, TCF7, LEF1), accompanied by corresponding upregulation and downregulation of transcriptional activity (77). Epigenetic mechanisms have been shown to influence cell-fate determinations in lymphocyte ontogeny (78, 79). Chromatin organization and histone-modifying enzymes represent critical regulators of dendritic cell (DC) functions. For instance, the histone H3K4 demethylase KDM5B exerts negative regulation on the activation of bone marrow-derived dendritic cells, leading to suboptimal T cell responses. Conversely, the methylated DNA “reader” methyl-CpG-binding domain protein 2 (MBD2) is indispensable for the phenotypic activation of dendritic cells and their capacity to prime T cell responses. Moreover, in-depth analysis of enhancer signatures like H3K4me1 and H3K27Ac has demonstrated that enhancers undergo substantial dynamics during T cell activation (80).

### 3.5 RNA modification

RNA modifications are chemical marks on the bases or riboses of RNA molecules. These modifications are widely present in various types of RNA (such as mRNA, tRNA, rRNA, lncRNA, etc.). To date, more than 150 different modifications have been identified, including methylation (e.g., m^6^A, m^5^C, m¹A), pseudouridylation (Ψ), acetylation, and so on. RNA modifications do not alter the sequence of RNA, but can dynamically regulate gene expression by affecting the structure, stability, translation efficiency of RNA and its interaction with other molecules. RNA modifications play a crucial role in the process of tumor immune evasion, reshaping the tumor microenvironment and interfering with immune surveillance mechanisms through multi-dimensional regulation (81, 82). For instance, in GBM, the long non-coding RNA CASC9 is involved in tumor progression by promoting glycolytic metabolism and is significantly associated with poor prognosis in patients. The underlying molecular mechanism involves regulation by N6-methyladenosine (m^6^A) modification: the m^6^A “reader” IGF2BP2 can recognize and bind to methylated CASC9, maintaining its function by enhancing the stability of its transcript. The molecular complex formed by these two further acts on the methylated region of hexokinase 2 (HK2) mRNA, directly driving glycolysis in GBM cells by improving the stability of this mRNA. The accumulation of large amounts of lactic acid resulting from enhanced glycolysis acidifies the tumor microenvironment (TME), thereby inducing immunosuppressive effects: the acidic environment not only impairs the proliferation, activation, and cytotoxic functions of immune effector cells such as T lymphocytes and natural killer (NK) cells, but also promotes the differentiation and expansion of regulatory T cells (Tregs). Ultimately, this inhibits systemic immune responses and facilitates tumor immune evasion (83, 84). A separate investigation revealed that targeting the m6A demethylase ALKBH5 disrupts YTHDF2-mediated stability of ZDHHC3 mRNA. This mechanism inhibits PD-L1 expression in gliomas by promoting PD-L1 degradation (85). Another study on immune evasion in gliomas demonstrated that the 28S rRNA methyltransferase NSUN5, via its cysteine 359 (C359)-dependent methyltransferase activity, directly binds to the chromatin-associated RNA (caRNA) of CTNNB1 and deposits 5-methylcytosine (m5C). This modification is oxidized to 5-hydroxymethylcytosine (5hmC) by TET2, which relies on TET2’s binding affinity for Fe²^+^ and α-KG. Subsequently, 5hmC is recognized by RBFOX2, a 5hmC-specific reader, which promotes the degradation of caRNA. This process downregulates β-catenin, enhances the phagocytic activity of tumor-associated macrophages (TAMs), and impairs the tumor’s ability to evade immunity (86).

## 4 Clinical implications and therapeutic strategies

The epigenetic panorama of gliomas reveals multiple modifications associated with tumor characteristics. Among gliomas, frequent occurrences include alterations in DNA methylation patterns, histone methylation/acetylation states, and variations in IDH mutation status. Strategies targeting the glioma epigenome are effective in tumor control and a valuable alternative. In glioma cells, epigenetic mechanisms have the ability to regulate immune responses, indicating that therapeutic approaches directed at epigenetic pathways may boost anti-tumor immune capabilities. Currently, several approaches targeting epigenetic alterations are in clinical trials: one targets mutated IDH with small molecule inhibitors and uses it for vaccination in relevant patients; another targets epigenetic modifiers like BET inhibitors (BETi), HDACi, DNMTi, and EZH2 inhibitors (EZH2i). **Table 1** summarizes the corresponding clinical trials on clinical-trials.gov.

**Table 1:** Ongoing clinical trials testing epigenetic drugs in glioma.

Inhibitor Category	Drug Name	Target	Clinical Trial Identifier	Trial Phase	Indication	Additional Notes	Pharmacokinetics & CNS Penetration	Efficacy Endpoints (Imaging & Survival)	Biomarker - Guided Patient Selection	Combination Strategies
IDH	AG - 881	Mutant IDH1 and/or Mutant IDH2	NCT02481154	Phase I	Glioma with IDH1 and/or IDH2 mutation	Dual - target inhibitor for IDH1 and IDH2 mutations	Preclinical data demonstrate effective blood - brain barrier (BBB) penetration; achieves therapeutic concentrations in glioma - bearing brain parenchyma in animal models	Imaging: MRI - based assessment of enhanced lesion regression rate, non - enhancing lesion changes; Survival: Progression - Free Survival (PFS), Overall Survival (OS); 12 - month PFS rate as a key secondary endpoint	Selected by IDH1/2 mutation status; prioritized for G - CIMP - positive tumors (frequent co - occurrence with IDH mutations); MGMT methylation status assessed to inform prognosis and combination potential	Exploration of combination with PD - 1/PD - L1 inhibitors to synergistically activate anti - tumor immunity; or with anti - angiogenic agents (e.g., bevacizumab)
IDH	AG - 120 (ivosidenib)	Mutant IDH1	NCT02073994	Phase II	Glioma with IDH1 mutation	Specific inhibitor for IDH1 mutations	Demonstrates BBB penetration; clinical data confirm measurable drug concentrations in brain tumor tissues	Imaging: Changes in tumor enhancement, tumor volume reduction rate; Survival: PFS, OS; 24 - month survival rate	Screened for IDH1 mutation; MGMT methylation status evaluated to predict temozolomide synergy potential; G - CIMP status for stratification	Combination with HDAC inhibitors (e.g., Vorinostat) to modulate epigenetic pathways; or with PD - 1 mAbs to enhance immune responses
IDH	AG - 221 (IDHIFA, Enasidenib)	Mutant IDH2	NCT02273739	Phase I/II	Glioma with IDH2 mutation	First - in - class IDH2 inhibitor	Limited BBB penetration; optimized dosing regimens (e.g., increased frequency) explored to enhance CNS exposure	Imaging: Tumor size changes, peritumoral edema evolution; Survival: PFS, OS; disease control duration	Selected by IDH2 mutation status and G - CIMP phenotype; MGMT methylation status referenced to evaluate immune microenvironment suitability	Combination with radiotherapy to enhance DNA damage effects; or with CTLA - 4 inhibitors to modulate immunity
IDH	BAY1436032	Mutant IDH1	NCT02746081	Phase I	Glioma with IDH1 mutation	Investigating safety and efficacy in early - phase trials	Preclinical glioma models verify CNS distribution; preliminary pharmacokinetic data support effective intracerebral concentrations	Imaging: Objective Response Rate (ORR) by neuro - oncology criteria; Survival: PFS, OS; early - stage treatment survival benefits	Screened for IDH1 mutation and G - CIMP positivity; MGMT methylation status analyzed for stratification	Combination with PARP inhibitors (e.g., Olaparib) targeting DNA damage repair pathways; or with PD - L1 mAbs
IDH	DS - 1001b	Mutant IDH1	NCT03030066	Phase I	Glioma with IDH1 mutation	Evaluating therapeutic potential in early - stage patients	Preclinical data confirm BBB penetration; intracerebral drug concentrations sufficient to inhibit IDH1 mutant enzyme activity in glioma models	Imaging: Tumor volume changes, new lesion occurrence; Survival: PFS, OS; quality - of - life - related survival indicators	Selected by IDH1 mutation and G - CIMP phenotype; MGMT methylation status assessed for prognosis	Exploration of combination with IDH peptide vaccines to synergistically activate specific immunity; or with PD - 1 blockers
IDH	IDH305	Mutant IDH1	NCT02381886	Phase I	Glioma with IDH1 mutation	Exploring efficacy across different glioma grades	Exhibits CNS - penetrating properties; animal experiments verify uniform intracerebral drug distribution	Imaging: Tumor regression rate stratified by glioma grade (low - grade, high - grade, anaplastic); Survival: PFS, OS by grade	Selected by IDH1 mutation and G - CIMP positivity; MGMT methylation status analyzed for grade - specific effects	Combination with radiotherapy to synergistically inhibit tumor proliferation; or with tumor metabolism - targeting agents
IDH	IDH305	Mutant IDH1	NCT02977689	Phase II	Grade II or III Glioma with IDH1 mutation	Focused on intermediate - grade gliomas	Same as IDH305 (NCT02381886)	Imaging: Tumor control rate (stability/reduction) in low - grade (II/III) gliomas; Survival: PFS, OS; progression - free survival extension	Selected by IDH1 mutation and G - CIMP positivity; MGMT methylation status evaluated for combination therapy impact	Combination with cytotoxic chemotherapy (e.g., temozolomide); or with immunomodulators (e.g., interferon - 纬)
IDH	IDH1R132H Peptide Vaccine	IDH1R132H Mutation	NCT02454634	Phase I	Grade III Glioma with IDH1 mutation	Immunotherapy approach for high - grade gliomas	Vaccine - mediated CNS action relies on antigen - presenting cell (APC) uptake and migration; no small - molecule - like "penetration" but requires efficient APC antigen presentation	Imaging: Differentiation of immune - related pseudoprogression, assessment of tumor enhancement changes; Survival: OS, PFS; duration of immune response	Selected by IDH1R132H mutation, HLA matching (for optimal antigen presentation); G - CIMP and MGMT methylation status assessed to evaluate the immune microenvironment	Combination with PD - 1 blockers to enhance tumor - specific T - cell responses; or with adoptive T - cell therapy
IDH	IDH1R132H Peptide Vaccine	IDH1R132H Mutation	NCT02193347	Phase I	Recurrent Grade II Glioma with IDH1 mutation	Targeting recurrent tumors with immunotherapy	Same as IDH1R132H Peptide Vaccine (NCT02454634)	Imaging: Tumor control (stability/reduction) in recurrent low - grade gliomas; Survival: PFS, OS; post - recurrence survival time	Selected by IDH1R132H mutation in recurrent gliomas; immune status (e.g., T - cell infiltration) and G - CIMP analyzed	Combination with CTLA - 4 inhibitors; or with dendritic cell vaccines to enhance immunity
IDH	IDH1R132H Dendritic Cell	IDH1R132H Mutation	NCT02771301	Safety Study	Glioma with IDH1 mutation	Assessing safety and immunogenicity	Based on dendritic cell (DC) re - infusion; DC migration to the CNS drives immunity; no small - molecule pharmacokinetic parameters	Imaging: Preliminary assessment of tumor imaging stability; Survival: Short - term safety - oriented follow - up, with subsequent focus on OS, PFS	Selected by IDH1R132H mutation; immune function availability (e.g., DC collectability and activation); G - CIMP and MGMT methylation status assessed	Combination with PD - L1 mAbs to enhance DC - mediated immune killing after antigen presentation
EZH2	Tazemetostat	Mutant and Wild - type EZH2	NCT03155620	Phase II	Glioma with EZH2, SMARCB1, or SMARCA4 mutation	Broad - spectrum inhibitor targeting multiple mutations	BBB - penetrating; preclinical models show CNS drug concentrations sufficient to inhibit EZH2 - related epigenetic regulation	Imaging: Tumor volume changes, enhancement alterations; Survival: PFS, OS; 6 - month progression - free rate	Selected by EZH2 mutation status; G - CIMP - related phenotypes (frequent association with some EZH2 mutations) analyzed; MGMT methylation status assessed	Combination with PARP inhibitors (e.g., Talazoparib) targeting DNA damage repair; or with PD - 1 blockers to modulate immunity
HDAC	Valproic Acid	Class I and II HDAC	NCT03243461	Phase III	Pediatric Glioma	Long - term study in pediatric patients	Exhibits CNS penetration; widely distributed in the brain, clinically used for neurological disorders	Imaging: Tumor control rate (stability/reduction) in pediatric gliomas; Survival: OS, PFS; long - term quality - of - life indicators for pediatric patients	Selected by pediatric glioma type (low - grade/high - grade); G - CIMP (if applicable) and MGMT methylation status (if present) analyzed	Combination with temozolomide (classic chemotherapy synergy); or with immunomodulators (e.g., interleukin - 2)
HDAC	Entinostat	Class I HDAC	NCT02780804	Phase I	Recurrent Childhood Visual Pathway Glioma	Investigating in rare pediatric brain tumors	BBB - penetrating; verified in pediatric brain tumor models	Imaging: Tumor regression in visual pathway gliomas, relief of optic nerve compression; Survival: PFS, OS; vision - preservation - related indicators	Selected by HDAC abnormalities, abnormal histone acetylation levels in childhood visual pathway gliomas; MGMT methylation status (if tested) analyzed	Combination with radiotherapy (synergistic DNA damage); or with neurotrophic agents (e.g., mecobalamin) to preserve visual function
HDAC	Panobinostat (LBH589)	Class I, II, and IV HDAC	NCT02717455	Phase I	Diffuse Intrinsic Pontine Glioma	Studying in difficult - to - treat pediatric brain tumors	Limited BBB penetration; intrathecal injection explored to enhance CNS concentrations	Imaging: Tumor stability in Diffuse Intrinsic Pontine Glioma (DIPG); Survival: OS, PFS; median survival time in DIPG	Selected by DIPG molecular features (e.g., H3K27M mutation); G - CIMP (if relevant) and MGMT methylation status (if present) analyzed	Combination with temozolomide (epigenetic regulation + chemotherapy); or with radiotherapy to enhance DNA damage effects
HDAC	Vorinostat (SAHA)	Class I and II HDAC	NCT01189266	Phase I/II	Diffuse Intrinsic Pontine Glioma	Multiple trials exploring its potential in pediatric gliomas	BBB - penetrating; preclinical and clinical data support effective intracerebral concentrations	Imaging: Tumor volume changes, enhancement in DIPG; Survival: OS, PFS; survival improvement in DIPG	Selected by HDAC abnormalities, H3K27M mutation in DIPG; G - CIMP and MGMT methylation status analyzed	Combination with immune checkpoint inhibitors (e.g., Atezolizumab) to modulate the tumor immune microenvironment
HDAC	Vorinostat (SAHA)	Class I and II HDAC	NCT02420613	Phase I	Diffuse Intrinsic Pontine Glioma	Continued investigation in pediatric brain tumors	Same as Vorinostat (NCT01189266)	Imaging: Radiological response (reduction/stability) in DIPG; Survival: OS, PFS; survival time extension trend in DIPG	Selected by DIPG molecular phenotype (e.g., H3K27M); G - CIMP - related features analyzed; MGMT methylation status assessed	Combination with drugs targeting H3K27M (if under development); or with radiotherapy
HDAC	Vorinostat (SAHA)	Class I and II HDAC	NCT00268385	Phase I	Glioma	Evaluating across different glioma types	BBB - penetrating; applicable for CNS treatment of multiple glioma types	Imaging: ORR, tumor volume changes across different glioma types; Survival: OS, PFS; survival differences by glioma type	Selected by HDAC abnormalities, G - CIMP positivity (in some gliomas); MGMT methylation status stratified	Combination with anti - angiogenic agents (e.g., Ramucirumab); or with PD - 1 blockers
HDAC	Vorinostat (SAHA)	Class I and II HDAC	NCT01236560	Phase I/II	Pediatric High Grade Glioma	Assessing efficacy and safety in pediatric patients	Same as Vorinostat pharmacokinetics	Imaging: Tumor regression rate, new lesion occurrence in pediatric high - grade gliomas; Survival: OS, PFS; quality of life in pediatric patients	Selected by IDH mutation (if present), G - CIMP phenotype in pediatric high - grade gliomas; MGMT methylation status assessed	Combination with adoptive T - cell therapy; or with tumor vaccines
HDAC	Vorinostat (SAHA)	Class I and II HDAC	NCT00555399	Phase I/II	Glioblastoma	Exploring in adult high - grade gliomas	BBB - penetrating; verified in adult glioblastoma (GBM) studies	Imaging: ORR in GBM by RECIST criteria, progression - free imaging; Survival: OS, PFS; 1 - year survival rate in GBM	Selected by G - CIMP positivity (in some GBM), MGMT methylation status (to predict temozolomide synergy), IDH mutation (if present)	Combination with temozolomide + radiotherapy (Stupp protocol - based synergy); or with PD - 1 mAbs
HDAC	Belinostat	Class I and II HDAC	NCT02137759	Phase II	Glioblastoma	Focused on adult patients with advanced gliomas	BBB - penetrating; clinical data show intracerebral drug concentrations in adult GBM	Imaging: Tumor volume changes, enhancement control in advanced GBM; Survival: OS, PFS; second - line treatment survival benefits in GBM	Selected by HDAC abnormalities, MGMT methylation status (to determine temozolomide combination potential), G - CIMP - related features	Combination with anti - PD - 1/PD - L1 agents; or with PARP inhibitors
HDAC	Vorinostat (SAHA)	Class I and II HDAC	NCT00731731	Phase I/II	Glioblastoma	Multiple trials in adult with advanced gliomas	Same as Vorinostat pharmacokinetics	Imaging: Response rate, progression - free imaging in advanced GBM; Survival: OS, PFS; survival associated with subsequent treatments in GBM	Selected by MGMT methylation status (methylation - positive prioritized for temozolomide combination), G - CIMP status, IDH mutation (if present)	Combination with bevacizumab (anti - angiogenic + epigenetic regulation); or with PD - 1 blockers
DNMT	5 - Azacytidine (Vidaza)	DNMT	NCT02940483	Phase I	Recurrent Posterior Fossa Ependymoma	Investigating in recurrent pediatric brain tumors	BBB - penetrating; verified in pediatric posterior fossa ependymoma models	Imaging: Tumor volume changes, enhancement in recurrent posterior fossa ependymoma; Survival: OS, PFS; post - recurrence survival time in ependymoma	Selected by DNMT abnormalities, ependymoma molecular subtype (e.g., RELA fusion); MGMT methylation status (if tested) analyzed	Combination with radiotherapy (synergistic DNA demethylation + damage); or with immunomodulators (e.g., granulocyte - macrophage colony - stimulating factor)
DNMT	5 - Azacytidine (Vidaza)	DNMT	NCT03020601	Phase I/Ib	Recurrent Ependymoma	Early - phase study in recurrent brain tumors	Same as 5 - Azacytidine (NCT02940483)	Imaging: Tumor control rate (stability/reduction) in recurrent ependymoma; Survival: OS, PFS; post - recurrence survival improvement in ependymoma	Selected by DNMT abnormalities, molecular markers (e.g., C11orf95 - RELA fusion) in recurrent ependymoma; G - CIMP (if relevant) analyzed	Combination with chemotherapy (e.g., carboplatin); or with agents targeting fusion genes (if available)
DNMT	5 - Azacytidine (Vidaza)	DNMT	NCT02223052	Phase I	Glioblastoma	Evaluating in adult glioblastoma patients	BBB - penetrating; used in adult GBM studies	Imaging: Tumor volume changes, new lesion occurrence in GBM; Survival: OS, PFS; 1 - year survival rate in GBM	Selected by MGMT methylation status (to predict temozolomide synergy), G - CIMP positivity (in some GBM), IDH mutation (if present)	Combination with temozolomide + PD - 1 blockers (epigenetic + chemotherapy + immunotherapy); or with radiotherapy

Source, https://clinicaltrials.gov.

### 4.1 Mutant IDH inhibitors

The aim of mutant IDH inhibitors is to block the synthesis of the oncometabolite 2-hydroxyglutarate (2HG). As a rival inhibitor of α-ketoglutarate-dependent dioxygenases, 2HG regulation can be achieved by suppressing its production, thereby reversing DNA hypermethylation and promoting the differentiation of mIDH1 glioma cells. Several mIDH1 inhibitors have shown effectiveness in both laboratory and animal models. Combining current first-line therapies (radiotherapy and temozolomide) with mIDH1 inhibitors and PD-L1-targeted immune checkpoint inhibitors (ICI) has enhanced tumor shrinkage in mIDH1 glioma-bearing mice, alleviated T cell exhaustion, and supported the formation of memory CD8+T cells. Presently, multiple clinical trials are assessing mIDH1 inhibitors for glioma treatment, although these remain in initial stages primarily focused on evaluating safety and the ability to reduce 2HG accumulation (87).

### 4.2 EZH2 inhibitors

Enhancer of zeste homolog 2 (EZH2), a pivotal histone methyltransferase in the PRC2, undergoes alterations in gliomas that lead to both gain- and loss-of-function mutations. These aberrations in EZH2 expression impact gene regulation by interacting with promoters and modulating methylation patterns, thereby acting as an oncogenic driver. EZH2-dependent silencing of TSGs drives oncogenic proliferation, aggressive invasion, and refractory resistance in cancer. The use of EZH2 inhibitors (EZH2is) has been shown to upregulate p16 expression and effectively curb the progression of gliomas. Research indicates that EZH2 serves as a pivotal regulator in regulating cancer cell immune response and mediates escape by downregulating immune activation genes, upregulating checkpoints, and creating an immunosuppressive TME. Tazemetostat, an EZH2i, is being tested in pediatric gliomas. However, A recent investigation indicates that it might influence the proliferation of primary Histone H3 Lysine 27 to Methionine Mutation (H3K27M)-mutant glioma cells occurs when functional p16INK4A is expressed in GBM cells harboring wild-type H3 and IDH (88). Short-term reduction of EZH2 correlates with decreased cell proliferation. However, recent findings show that extended EZH2 inhibition might induce a cell fate transition, promoting both proliferation and DNA damage repair mechanisms, which ultimately drives tumor progression (89).

### 4.3 DNA methyltransferase inhibitors

Epigenetic silencing by DNMTs suppresses gene expression via CpG island methylation. DNMTis reverse this, reactivating tumor suppressors and inducing anti-tumor effects. In gliomas, DNMTis upregulate MHC class I presentation, enhancing CD8+ T cell recognition. By preventing DNMT-driven epigenetic reprogramming, DNMTis also counteract T cell exhaustion. Genetic knockout of DNMT3A in CAR T cells mirrors this effect, bolstering anti-tumor immunity (50, 90). Preclinical research has shown DNMTi exhibit efficacy using *in vitro* and *in vivo* model systems of IDH-mutant (IDHmt) gliomas. Nevertheless, this preclinical promise has not yet led to successful clinical outcomes for glioma patients treated with DNMTi, potentially due to the S-phase specificity and relatively brief half-life of agents like 5-azacytidine and decitabine. Nevertheless, whether gliomas require general demethylation remains controversial, activation of proto-oncogenes coupled with hypomethylation-mediated reactivation of the DNA repair gene O^6^- methylguanine-DNA MGMT might confer resistance of glioblastoma to alkylating agents. Notably, low-dose demethylating agents can modulate immunity and potentially trigger an innate immune response through reactivation of retroviruses (91).

### 4.4 Histone deacetylase inhibitors

Certain frequent epigenetic alterations in neoplastic cells involve dysregulation of histone modifications within oncogene/tumor suppressor gene regulatory domains. Aberrant HDAC expression in cancer cells modifies the cell cycle and initiates tumorigenesis. HDAC expression patterns correlate with glioma malignancy grading; class II and IV HDAC isoforms exhibit reduced expression in GBM compared to low-grade astrocytomas. HDAC1 is excessively expressed in multiple glioma subtypes and associated with diminished overall survival. HDACis modulate antitumor immune responses by facilitating T cell chemokine production, augmenting PD-1-targeted immunotherapy effectiveness, and increasing PD-L1 and HLA-DR surface expression on tumor cells, suggesting a potential cooperative effect between HDACis and ICIs for glioma therapy. Vorinostat, evaluated in a phase II clinical trial for recurrent GBM, showed favorable tolerability as monotherapy and influenced GBM-relevant signaling pathways. Nevertheless, its clinical application is constrained by toxicity and suboptimal efficacy, potentially attributed to inadequate BBB penetration. Integrating HDACis with other therapeutic approaches or enhancing BBB permeability could improve treatment outcomes (92–94).

### 4.5 BET inhibitors

BRD4 function can be effectively suppressed by inhibitors or degraders. As small-molecule compounds, BRD4 inhibitors have the potential to augment cancer therapy by mimicking acetyl-lysine residues, thereby binding to BET proteins and ultimately interfering with the interaction between BET proteins, acetylated histones, and transcription factors (95). BET proteins play a critical role in driving high-level oncogene expression. BETi can attenuate oncogene transcription by diminishing super-enhancer activity, which consists of large aggregations of transcriptional enhancers that govern gene expression in cell identity and pathological conditions like cancer. Using *in vivo* RNA interference screening to target chromatin regulators critical for GBM cell viability, BRD4 emerged as a prominent target. Preclinical investigations using orthotopic mouse glioblastoma xenograft models have demonstrated the efficacy of multiple BETi compounds, such as JQ1, I-BET151, and OTX015 (96). Primary glioma cells with IDH mutation (IDHmt) demonstrate significant sensitivity to BET inhibitors JQ1 and GS-626510, exhibiting half-maximal inhibitory concentrations (IC50) 1,000 times lower than that of Temozolomide. A 2015 phase IIa clinical trial evaluating OTX015 for dose optimization in recurrent glioblastoma patients was halted due to insufficient therapeutic response. Alternative BET inhibitors featuring distinct pharmacokinetic properties are currently under preclinical assessment (97, 98).

### 4.6 Antigen-specific CAR T-cell immunotherapy

Chimeric antigen receptor (CAR)-modified T lymphocyte adoptive transfer emerges as a promising immunotherapeutic approach for central nervous system malignancies. Using genetic modification methods, autologous T cells are reprogrammed to display synthetic antigen receptors on their cellular membranes, enabling targeted recognition of tumor-associated antigens (61, 99). These genetically engineered CAR-T cells exhibit the ability to selectively identify and attach to neoplastic cells, subsequently triggering the immune-effector function of T lymphocytes to efficiently eradicate neoplastic cells (100). Actually, the success of this strategy relies on the recognition of relatively specific cancer targets on the cell surface, effectively overcoming the common immune evasion mechanisms adopted by tumor cells. Clinical investigations into CAR-T cell therapy for glioblastoma have devoted to five distinct antigens: EphA2, EGFRvIII, HER2, IL13Rα2, and GD2. The early results of these targets have been published, revealing some remarkable achievements (101, 102). Although CAR T cell therapy didn’t improve OS significantly, one glioblastoma patient survived 59 months after EGFRvIII-CAR T cell therapy without post-CAR treatment, and some on HER2-CAR T cell therapy had stable disease up to 29 months. A patient treated with anti-IL13Rα2 CAR T cells achieved a full remission within 7.5 months, showing potential.

**Figure 5:**
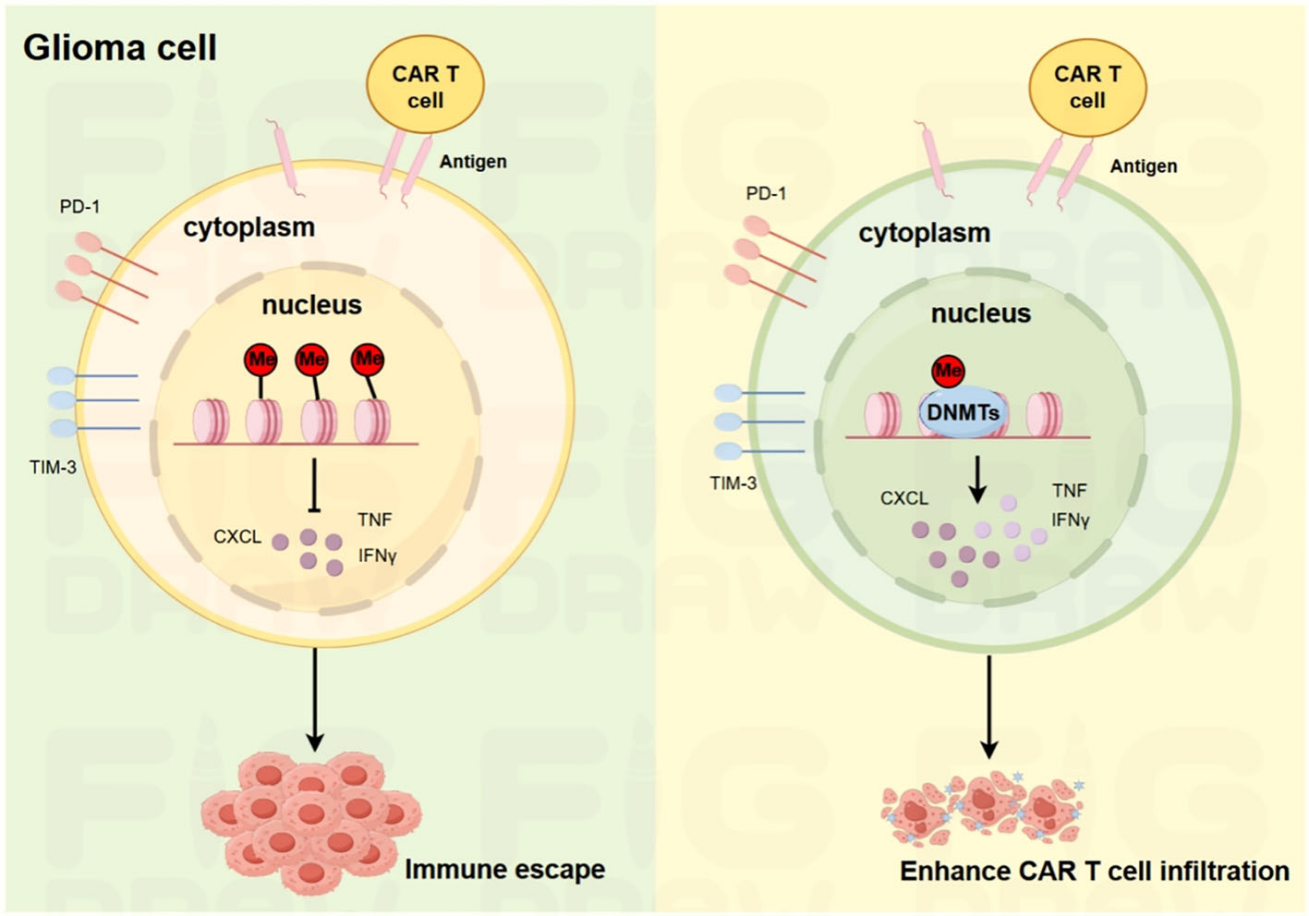
Schematic illustration of the mechanisms underlying glioma immune escape and enhanced CAR-T cell infiltration. On the left, in glioma cells, the expression of PD-1 and TIM-3, along with the release of cytokines such as CXCL, TNF, and IFNγ, contributes to immune escape. On the right, the introduction of DNMT in CAR-T cells modulates the cellular environment, reducing immune-suppressive factors and enhancing the infiltration ability of CAR-T cells into glioma tissues.

However, tumor cell immune evasion against CAR T cells and inadequate CAR T cell trafficking to tumor loci represent primary challenges for CAR T cell therapy in solid tumor treatment, with patients frequently experiencing relapse following initial tumor regression. Thus, enhancing CAR T cell infiltration into tumor sites emerges as a critical strategy to circumvent this immune evasion (**Figure 5**). Studies have shown that upregulated chemokine receptor expression in CAR-T cells can boost their ability to infiltrate tumor microenvironments (103). For example, increased chromatin availability at the CD56 genomic region, together with chemokine upregulation including CXCL9, CXCL10, and CXCL12, enhances tumor infiltration by PD-1/TIM3/LAG3-modified CAR-T cells. Moreover, expression of CASTAT5 (a constantly active STAT5 isoform) fosters CD4+ T cell infiltration and migration to tumor microenvironments through epigenetic reprogramming. In addition, T cell dysfunction and impaired trafficking arise when miR-155 directly inhibits suppressor of cytokine signaling 1, disrupting normal regulatory mechanisms (104); The upregulation of miR-155 in CAR T cells appears to be an interesting strategy to promote the transportation of CAR T cells and an effective anti-tumor response (105); Targeting let-7 miRNA, which suppresses CCR2/CCR5 expression in T cells, might improve CAR-T cell infiltration by restoring chemokine receptor activity (106). Latest studies also show CAR T cell activation, proliferation, and survival are regulated epigenetically (88). For example, DNMT3A (a DNA methyltransferase) regulates epigenetic changes during chronic LCMV infection’s effector-to-exhaustion transition. *In vivo*, DNMT3A-deficient CAR-T cells outperform wild-type ones, enhanced by IL-10; double KO CAR-T cells confirm this. SUV39H1 (a histone methyltransferase) modulates chromatin and gene expression, and disrupting it in CAR-T cell therapy boosts cell expansion, persistence, and efficacy. TET2 (a methylcytosine dioxygenase) promotes DNA demethylation. BATF3, a key regulator of memory T cell proliferation and differentiation, plays a critical role in T cell exhaustion—particularly in TET2-knockout CAR-T cells—underscoring the therapeutic possibility of epigenetic modulation in CD8+ T lymphocytes (107). Leveraging epigenetic modulation to boosting CAR-T cell effectiveness presents a promising clinical approach to address the current limitations of solid tumor treatment, where conventional CAR-T approaches have shown limited success. Combining epigenetic agents with adoptive cell therapy may overcome barriers by reprogramming the TME and enhancing T cell persistence.

## 5 Toward clinical translation: challenges and opportunities

Immunotherapy has emerged as a rapidly evolving field in glioma treatment, but it is fraught with multiple hurdles. Firstly, the post-treatment local immunosuppressive TME restricts therapeutic efficacy, yielding modest outcomes that only benefit a small subset of patients. Secondly, gliomas are characterized by a scarcity of distinct tumor antigens and significant intratumoral heterogeneity, complicating targeted immunotherapeutic approaches. Thirdly, the chronic immunotoxicity associated with immunotherapies and their long-term sequelae pose substantial concerns. Despite preclinical research and early-stage (I/II) therapeutic trials have produced promising data, and some individual cases have demonstrated success, advancing from phase II/III trials remains a formidable obstacle. Currently, there are no reported Phase III clinical trials that have revealed the efficacy of immunotherapy across large patient populations with glioma (108, 109).

Epigenetic modifications, ubiquitous in tumors, play a key role in establishing and maintaining the heterogeneity of glioblastoma (GBM). Aberrant epigenetic regulation is a major driver of GBM initiation, and dysregulation of epigenetic modulators promotes tumor formation. Epigenetic drugs such as DNMT inhibitors (e.g., 5-azacytidine and decitabine) have shown anti-tumor activity in preclinical GBM models and have been approved by the FDA for the treatment of various tumors (110, 111). However, the clinical efficacy of the EZH2 inhibitor tazemetostat in GBM remains controversial, and its specific benefits require careful evaluation (112, 113); HDAC inhibitors (HDACIs), on the other hand, hold potential as therapeutic agents by regulating oncogene transcription and cell cycle, among other processes (114). Recent studies have identified lactate-derived histone lactylation as a novel modification associated with GBM progression (115). Despite the great potential of epigenetic strategies in glioma treatment and the fact that multiple inhibitors have entered clinical trials, results from large-cohort trials have been suboptimal. Off-target effects, difficulties in crossing the blood-brain barrier, and tumor heterogeneity are the main reasons, making it imperative to optimize drug delivery and targeting strategies.

To overcome the therapeutic bottlenecks of applying epigenetic drugs in GBM, the integration of medicine and engineering has provided new ideas for drug delivery and targeting, such as zinc ionophores, leveraging their tissue specificity, are combined with CpG nanoparticles and AMD-Zn to construct injectable hydrogel systems (imGEL), which enhance drug efficacy and inhibit recurrence through the tissue affinity of zinc particles and the diffusion-retention properties of hydrogels (116); special hydrogel composites combined with GSC-specific CAR-macrophages, when injected into the tumor cavity after GBM resection in mice, exert strong immunotoxicity against tumors to suppress recurrence. In addition, direct intratumoral administration is highly effective: sonodynamic therapy (SDT) eradicates GBM cells by activating photosensitizers with ultrasound to generate reactive oxygen species and cavitation bubbles (117); oncolytic viruses (OVs), after genetic engineering to target tumor cell receptors, can trigger host immune responses to clear cancer cells. Advances in OV delivery technologies have overcome the blood-brain barrier, such as the convection-enhanced delivery of PVSRIPO reported by Desjardins et al., which targets GBM via CD155 (118). Preliminary data show that patients receiving PVSRIPO have higher survival rates than historical controls, and its phase II trial (NCT02986178) alone or in combination with lomustine is underway, with its efficacy in GBM patients highly anticipated (119).

CAR-T therapy succeeds in hematologic malignancies but fails in solid tumors partly due to CAR-T exhaustion, making combination strategies promising for gliomas (120). EZH2, a PRC2 component catalyzing H3K27me3, is key for T-cell functions. A study showed that resting exhausted CAR-T cells with EZH2 inhibitor tazemetostat (D11-15) remodeled exhaustion-related epigenome via EZH2, restoring function (120, 121). Another study identified class I HDACi (e.g., M344, chidamide) as CAR-T enhancers via screening 370 drugs, enhancing memory and anti-exhaustion to induce sustained anti-tumor effects. Mechanistically, HDACi activates Wnt/β-catenin pathway by regulating HDAC1, H3K27ac, and TCF4/LEF1/CTNNB1 (122). Additionally, DNMT3A (a major *de novo* methyltransferase) upregulates after TCR activation, mediating T-cell polarization via methylation. *De novo* methylation exacerbates T-cell exhaustion, while DNMT3A deficiency reduces methylation at key loci, enhancing anti-PD-L1 response. Low-dose decitabine pretreatment enhanced T-cell response in mice. In solid tumor (including glioma) CAR-T models, DNMT3A deficiency boosted CAR-T expansion, cytokine secretion, and lysis; after chronic stimulation, these cells showed higher TCF1/LEF1 and stem/naive-like epigenetics, increasing *in vivo* anti-tumor activity (123, 124).

CAR-T and epigenetic combination therapy offers hope for glioma treatment but faces challenges: glioma heterogeneity limits CAR-T recognition, the complex brain microenvironment inhibits CAR-T function, and patient variability demands personalized plans. However, it provides new insights, with deeper research potentially yielding precise strategies like optimized targets, epigenetic drug combinations, and biomarkers. A GO analysis linked DNMT3A-regulated genes to pathways like TCR signaling, proposing DNMT3A depletion signatures as biomarkers for patient selection and response prediction (123). Another study on pediatric high-grade CNS tumors noted variable B7-H3 expression (a potential marker for CAR-T), recommending tumor tissue testing during B7-H3-targeted trials to assess IHC B7-H3 as a biomarker for tailored treatment (125). Looking forward, single-cell and spatial epigenomic mapping will help address these challenges. They can capture CAR-T cell exhaustion trajectories, functional heterogeneity and epigenetic interactions with tumor cells, and analyze spatial correlations between regional tumor epigenetic landscapes, immune infiltration and CAR-T function. This will advance CAR-T-epigenetic combination therapy from “broad-spectrum exploration” to “precision matching”, supporting personalized treatment of gliomas and other solid tumors.
